# Differences in the Pathological Diagnosis and Repeat Craniotomy Rates in Cerebral Tumors Undergoing Biopsy or Resection in an Urban Versus Regional Center

**DOI:** 10.1097/MD.0000000000002131

**Published:** 2015-10-30

**Authors:** Craig R. Vonhoff, Alistair Lochhead, Stavros Koustais, Nicole Watson, Juliana Andrici, Janice Brewer, Anthony J. Gill

**Affiliations:** From the Department of Neurosurgery, Wollongong Hospital (CRV, SK); Southern IML Pathology, Wollongong (AL); Cancer Diagnosis and Pathology Research Group, Kolling Institute of Medical Research (NW, JA, AJG); Department of Anatomical Pathology, Royal North Shore Hospital, St Leonards (JB); Sydney Medical School, University of Sydney (JA, JB, AJG); and Sydney Vital Translational Research Centre, Royal North Shore Hospital, Pacific Highway, St Leonards, New South Wales, Australia (AJG).

## Abstract

Primary intracranial tumors occur with an incidence of between 2.5 and 6 per 100,000 individuals. They require specialist expertise for investigation and management including input from radiology, pathology, neurosurgery, and oncology. Therefore, most patients with intracranial neoplasia are investigated and managed in larger hospitals. The geographically dispersed population of Australia has facilitated the development of neurosurgical units in regional areas. However, major metropolitan hospitals are over-represented compared with regional centers in most research cohorts. We therefore sought to investigate the spectrum of intracranial neoplasms undergoing biopsy and surgery at a major regional center in Australia and to compare the demographic and pathological features to similar cohorts treated in major metropolitan hospitals.

We searched the pathological databases of both a major regional pathology provider and a major metropolitan pathology practice, which provides surgical pathology services for both a large private and a large public neurosurgical hospital, to identify all cerebral tumors undergoing biopsy or resection over a 14-year period (calendar years 2001 and 2014).

In all, 3717 cerebral tumors were identified. Among them, 51% were from an urban private hospital, 33% from an urban public hospital, and 16% from a regional public hospital. Overall, one-third of them were neuroepithelial in origin, a quarter metastatic disease, a fifth meningeal, and one-tenth were pituitary adenomas. The regional center treated a higher proportion of metastatic tumors and less meningeal tumors compared with the urban center. Additionally, patients were less likely to undergo a second operation in the regional center (*P* < 0.001). The differences give an important insight into the burden of neurosurgical disease in regional Australia, and how it differs from that encountered in large metropolitan centers.

## INTRODUCTION

Intracranial neoplasms are an important cause of morbidity and mortality. The incidence of primary malignant brain tumors globally is 2.6 and 3.7 per 100,000 in females and males, respectively. In developed countries alone, this figure is almost double—4.1 to 5.8 per 100,000.^1^ In Australia, malignant primary brain tumors are the 13th most common malignancy, and the 10th most common cause of cancer death. The overall 5-year survival is 22% compared with 66% for all cancers, and they account for the third longest average length of stay for hospital admissions due to malignancy (12 days).^2^ Intracranial metastatic disease is also a significant cause of morbidity and mortality, with a median survival ranging from 3 to 18 months post-treatment.^3,4^

An increase in the number of neuro-oncology centers in Australia, coupled with population growth both in regional and metropolitan areas, has coincided with significant advancements and innovations in neuro-oncology. Whereas the overall incidence^5^ and survival^6^ of primary brain tumors in Australia has been reported, to date, there are little data addressing the differences in presentation and management of primary cerebral neoplasia at major urban compared with regional centers.

We therefore sought to audit the experiences of a major Australian regional neurosurgical center and compare the incidences of different types of cerebral neoplasia undergoing biopsy or resection in this rural/regional center with a major metropolitan public and private hospital.

## METHODS

We searched the computerized databases of 2 separate public hospital anatomical pathology laboratories in New South Wales (NSW), Australia, to identify all cases of cerebral neoplasia undergoing surgical resection, form calendar year 2001 to 2014. One of these laboratories provided all surgical pathology services for a regional neurosurgical unit, whereas the other serviced the neurosurgical unit of both a major capitol city public and private hospital.

The demographic and pathological features of all confirmed cases of cerebral neoplasia were recorded. Primary intracranial neoplasms were then classified according to the 2007 World Health Organization (WHO) classification of central nervous system tumors into neuroepithelial tumors (which included all astrocytic, oligodendroglial, ependymal, mixed glial, choroid plexus, neuronal and mixed-neuronal-glial, pineal, neuroblastic, or glioblastic tumors), meningeal, or other subtypes of primary or metastatic neoplasms.^7^

Tumors that typically demonstrate an extracranial component such as chordoma and neuroblastoma were excluded, as were vascular lesions such as cavernous hemangioma and arteriovenous malformations. Repeated presentations of the same patient were identified and compiled before deidentification of data to enable calculation of tumor presentations and reoperation rates. The clinical and pathological characteristics between the different treating hospitals were compared using a chi-square test for differences in the frequency distributions of categorical study variables, and 1-way analysis of variance (ANOVA) to compare the means of the continuous variable of age. Ethical approval for this study (ref: LNR/13/WGONG/131) was provided by the University of Wollongong/Illawarra and Shoalhaven Local Health District Health and Medical HREC on October 28, 2014.

## RESULTS

A total of 3717 pathology specimens were identified over the study period, from 3268 brain tumors. The clinical, pathological, and demographic features are presented in Table 1. Briefly, 379 patients underwent a second operation either subsequent to a diagnostic biopsy, subtotal resection, or re-excision at a later date, equating to a reoperation rate of 11.2%. Of all tumors, 51.4% were from a city private hospital, totaling 1960 specimens from 1674 tumors; 33.0% of tumors were from a city public hospital totaling 1196 specimens from 1075 tumors; and 15.6% of tumors were from a regional public hospital totaling 561 specimens from 519 tumors. The reoperation rate was 13.9%, 10.0%, and 7.5%, respectively.

**TABLE 1:**
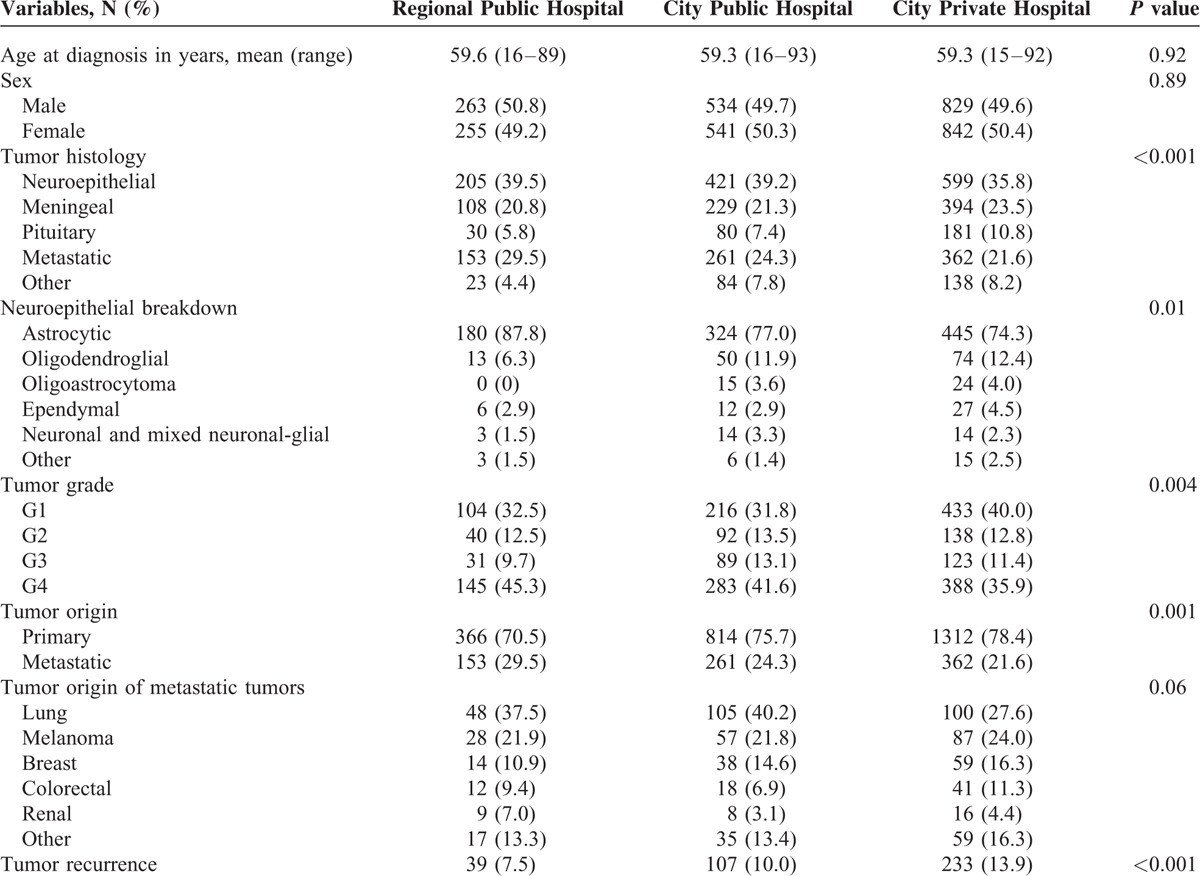
Clinical and Pathological Characteristics of 3268 Patients With Brain Tumors, Grouped by Treatment Hospital

### Capital City Private Hospital

Among the patients undergoing surgery at the major capital city private hospital, 49.6% were males, with an average age at initial surgery of 59.3 ± 15.1 years (range 15–93 years). Most neoplasms were neuroepithelial in origin (599; 35.8%), closely followed by meningeal (394; 23.5%), metastatic (362; 21.6%), and pituitary adenomas (181; 10.8%), as shown in Table 1. The spectrum of neuroepithelial neoplasms is also illustrated in Table 1. Briefly, the majority were astrocytic (445; 74.3%), with a much smaller proportion of oligodendroglial tumors (74; 12.4%) and few other subtypes. Most neuroepithelial tumors were WHO grade IV (383; 64.4%), with a serially decreasing proportion of grade III (114; 19.0%), grade II (62; 10.4%), and grade I (37; 6.2%) tumors. Most primary cerebral but non-neuroepithelial tumors were meningiomas, with the remainder comprised of hemangioblastomas (24), hemangiopericytomas (2), and solitary fibrous tumors (1). Two hundred and eighty-six (77.3%) meningiomas were WHO grade I, 75 (20.3%) were atypical (WHO grade II), and 9 (2.4%) were anaplastic (WHO grade III). The most common metastatic tumors were from the lung (100; 27.6%), melanoma (87; 24.0%), breast (59; 16.3%), and colorectal (41; 11.3%) origin. Among the metastatic tumors, 16.3% were of unknown primary site at the time of initial pathological diagnosis.

### Capital City Public Hospital

Among the patients operated on at the capital city public hospital, 49.7% were males averaging 59.3 ± 15.6 years old (range 16–93 years). Most tumors were neuroepithelial in origin (421; 39.2%) followed by metastatic disease (261; 24.3%), meningeal tumors (229; 21.3%), and pituitary adenomas (80; 7.4%), as shown in Table 1. Neuroepithelial tumors were largely astrocytic in origin (324; 77.0%), followed by oligodendroglial tumors (50; 11.9%) and others, as shown in Table 2. Most neuroepithelial tumors were WHO grade IV at diagnosis (283; 67.2%), followed by grade III (80; 19.0%), grade II (41; 9.7%), and grade I (17; 4.0%). Meningiomas were the most common non-neuroepithelial tumors and were comprised largely of WHO grade I (156; 77.3%), with a smaller collection of atypical WHO grade II (50; 20.3%) and anaplastic WHO grade III (7; 3.3%) tumors. The remainder of non-neuroepithelial tumors were hemangioblastomas (12), hemangiopericytomas (2), and solitary fibrous tumors (2). Intracranial metastasis in patients presenting to this city center public hospital originated largely from the lung (105; 40.2%), followed by melanoma (57; 21.8%), breast (38; 14.6%), and colorectal (18; 6.9%). Thirty-five (13.4%) metastatic tumors were of unknown origin at time of diagnosis (Table 1).

**TABLE 2:**
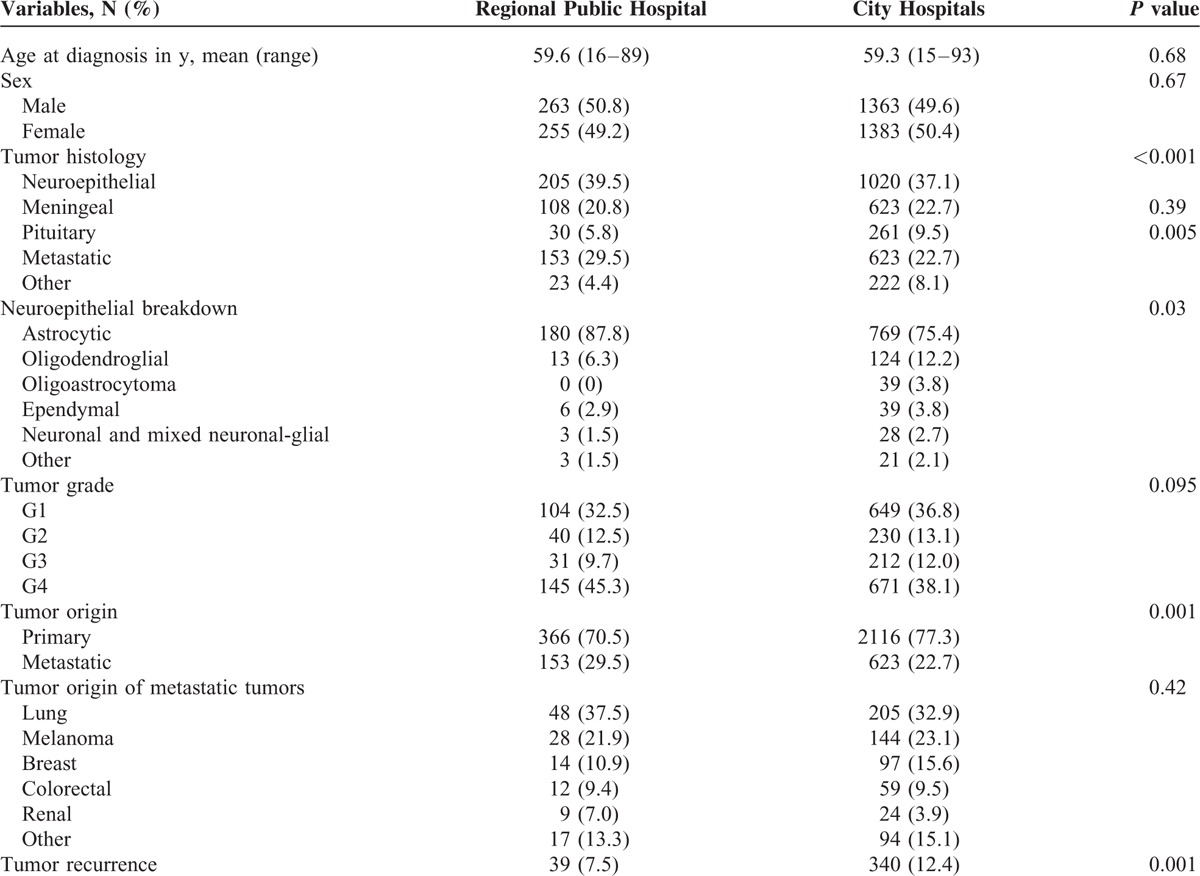
Clinical and Pathological Characteristics Comparison of Regional Public Hospital to City Hospitals (Both Private and Public)

### Regional Public Hospital

The regional hospital cohort was comprised of 50.8% males, and the average age at initial diagnosis was 59.6 ± 14.5 years (range 16–89 years). The tumor origin was neuroepithelial (205; 39.5%), metastatic disease (153; 29.5%), or meningeal (108; 20.8%), with a smaller number of pituitary adenomas (30; 5.8%), lymphomas (14; 2.7%), and others, as shown in Table 1. The majority of neuroepithlial tumors were astrocytic (180; 87.8%), with a prevalence of WHO grade IV (144; 66.4%), as shown in Table 2. Meningeal tumors were comprised of 104 meningiomas (96.2%), of which 91 (87.5%) were WHO grade I, 12 (11.5%) were WHO grade II, and 1 grade III. Three hemangioblastomas and 1 hemangiopericytoma were identified. Metastatic disease was most commonly from the lung (48; 37.5%), followed by melanoma (28; 21.9%), breast (14; 10.9%), and colorectal (12; 9.4%).

The clinical and pathological features of the 3 patient groups (regional public hospital, city public hospital, city private hospital) are compared in each of Tables 2–5.

**TABLE 3:**
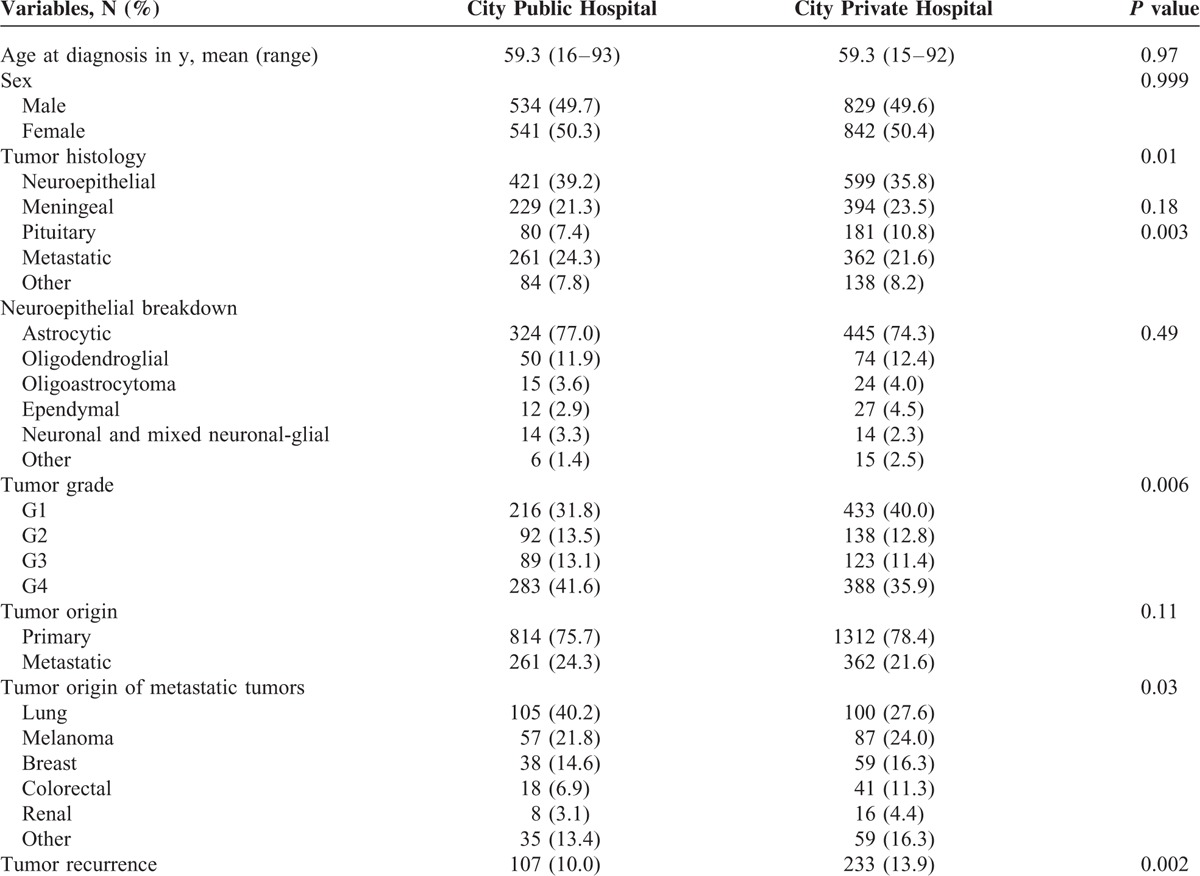
Clinical and Pathological Characteristics Comparison of City Public Hospital with City Private Hospital

**TABLE 4:**
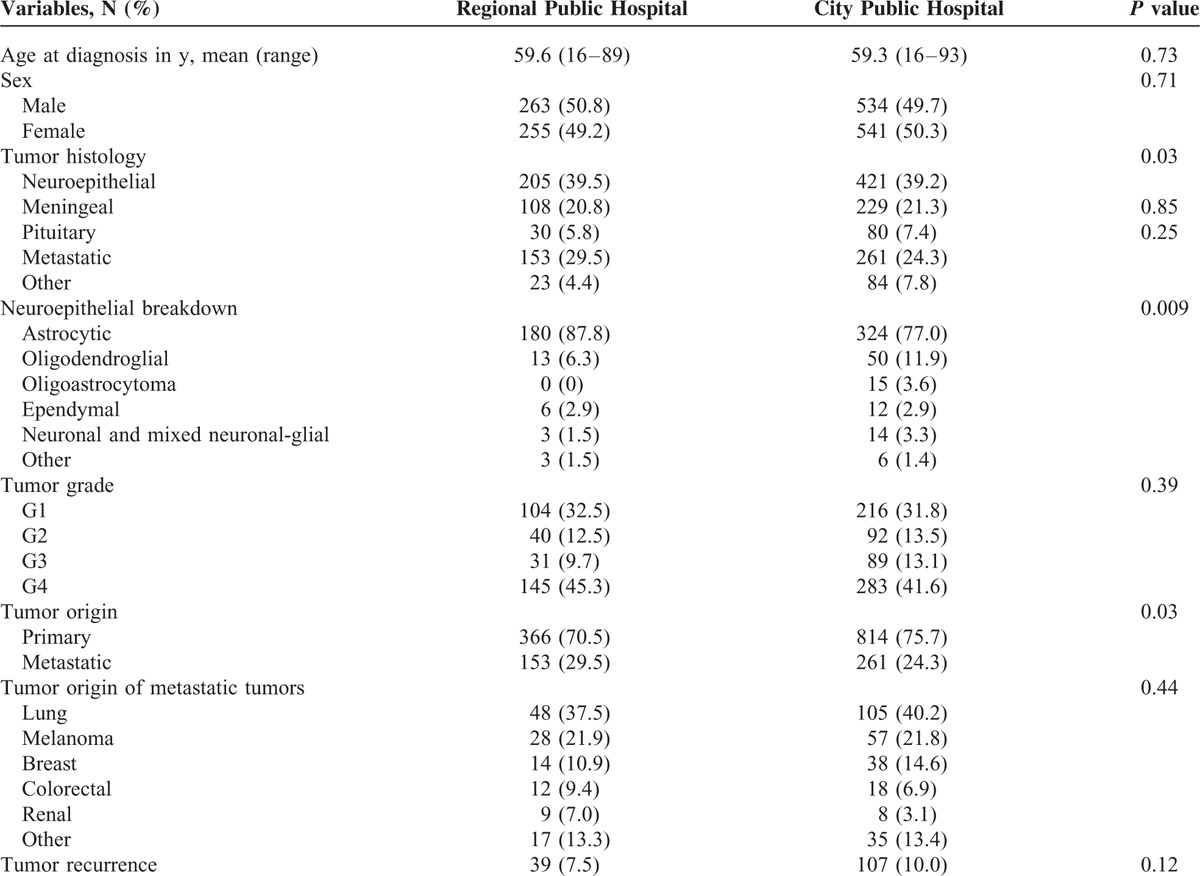
Clinical and Pathological Characteristics Comparison of Regional Public Hospital With City Public Hospital

**TABLE 5:**
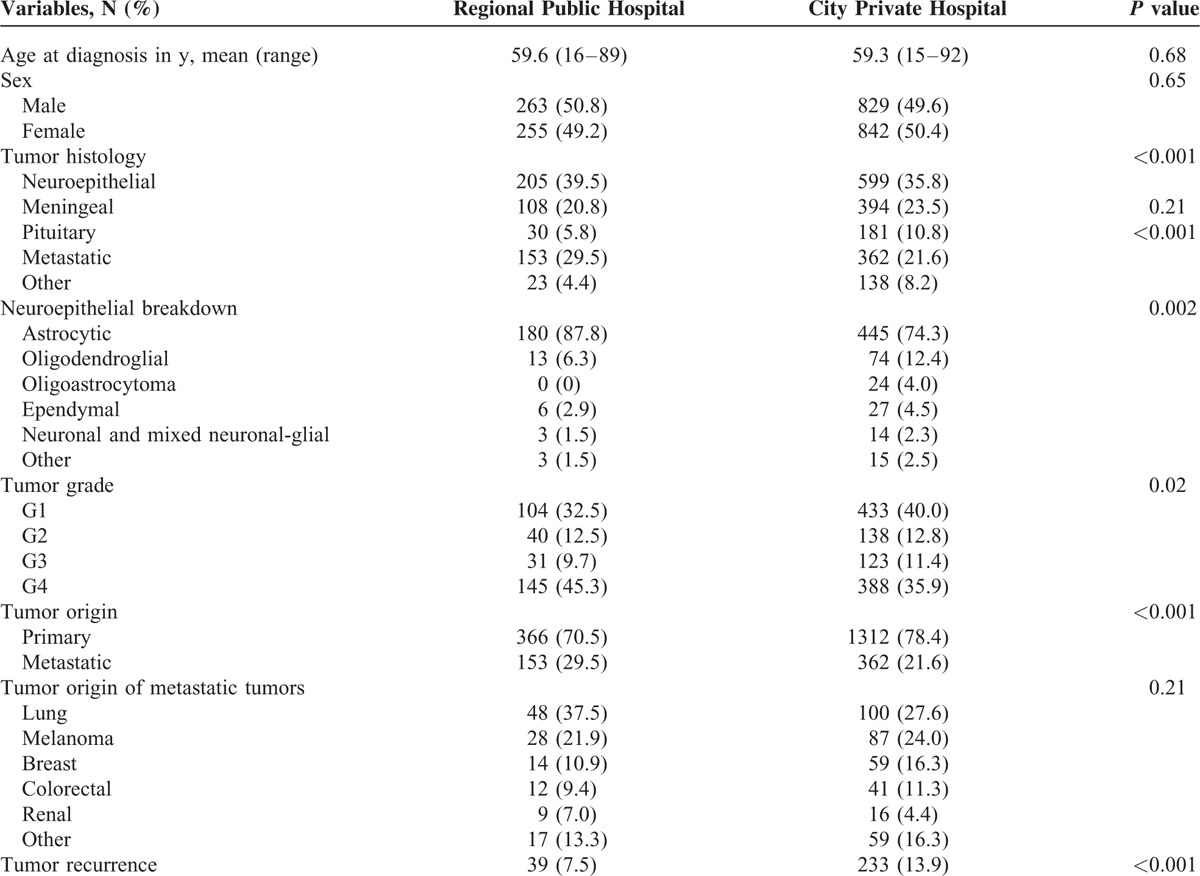
Clinical and Pathological Characteristics of Regional Public Hospital With City Private Hospital

## DISCUSSION

This study describes an audit of 3268 histologically defined intracranial neoplasms from 3 institutions over a 14-year period. Over a third of all intracranial neoplasms were neuroepithelial in origin, a quarter were of metastatic disease, a fifth meningeal, and 10% pituitary adenomas. Because this study was based on auditing the surgical pathology databases of 2 institutions, the population is limited to presentations that were both appropriate for surgical intervention and yielded tissue for histopathological diagnosis. Most neuroepithelial tumors were astrocytic in origin, with a significant component of tumors displaying oligodendroglial differentiation (11.2%). In addition, the vast majority were classified as WHO grade IV. The 3 most common origins of metastatic disease demonstrated in this population were lung, melanoma, and breast. A higher proportion of intracranial neoplasms treated in the regional center were metastatic tumors (29.5% vs 23% in urban centers), and lung and melanoma were the 2 most common origin of metastatic disease.

We accept that the survey of 3 hospitals may not be representative of trends across all regional and metropolitan hospitals; however, it is interesting to note significant differences in the spectrum of disease presenting to each institution. Overall, a larger proportion of primary brain tumors presented to city centers as compared with the regional center (*P* = 0.001). Neuroepitihelial tumors differed between groups in both histological subclassification (*P* = 0.01) and grade (*P* < 0.01). It is interesting to note that the difference in tumor grade was most notable between the city public and private hospitals. This may represent a referral bias towards the private system for patients with slow-growing disease, most of which is treated semielectively, or surgeon preference for treating less complex tumors in the private setting. In contrast, patients with high-grade lesions are more likely to present acutely and therefore undergo surgery in the public setting.

Tumors with oligodendroglial differentiation were less common in the regional center (6.3% of all neuroepithelial were oligodendrogliomas and there were no oligoastrocytomas) compared with the urban center (12.2% oligodendrogliomas and 3.8% oligoastrocytomas). The regional center currently does not support a specialist neuropathologist, and difficult cases historically have been referred externally for specialist review. Fluorescent in-situ hybridization (FISH) analysis for 1p/19q codeletion has been obtained with increasing frequency during the study period where oligodendroglial differentiation has been suspected. Overall, this was performed on 51% of glial tumors. Routine referral for FISH analysis of all glial tumors may aid in improving diagnostic accuracy.

Recognition of oligodendroglial differentiation has historically been a challenge in neuropathology, resulting in significant interobserver variability. It is well recognized that subspecialist neuropathologists are more likely to identify oligodendroglial differentiation than general surgical pathologists.^8–10^ In 1997, Coons et al^9^ found a program of joint review by multiple pathologists both improved intradepartmental concordance rates and increased the rate of diagnosis of oligodendroglial tumors. In addition, objective molecular criteria for classification of glial tumors would greatly benefit pathologists. A study by Reus et al^11^ in 2014 demonstrated a diagnostic algorithm using sequential alpha-thalassemia/mental retardation syndrome X-linked (ATRX) immunohistochemistry, isocitrate dehydrogenase 1 gene (IDH1) mutation, 1p/19q and subsequent copy number analysis to better classify glial tumors. Using the consensus ISH-Haarlem guidelines, they were able to classify adult glioma tumors as astrocytomas, oligodendrogliomas, and glioblastomas in a way they conclude is better associated with patient outcomes.

Improving the diagnostic reliability of neuroepithelial tumors is imperative because of the significant impact on treatment and prognosis in the diagnosis of oligodendroglial differentiation.^12,13^ This study adds weight to the need for more objective histological criteria for the grade and linage of gliomas.^14^

The number of patients undergoing multiple operations for the same tumor was different between the populations studied. Overall, 7.5% of cases underwent a second operation in the regional center, compared with 10.0% at the city public hospital and 13.9% at the city private hospital (*P* < 0.001). This trend was present for all tumor subtypes, except for pituitary adenomas and meningeal tumors. Rates of reoperation for intracranial metastases in particular were remarkably different, with 3.3% regional compared with 8.0% city public and 20.4% at city private hospitals. Published caseloads from other institutions describe reoperation rates somewhere in between these values. Indeed, in appropriately selected patients, repeat craniotomy for resection of intracranial metastatic disease is correlated with better outcomes.^15,16^ Reoperation rate for glioblastoma have previously been reported as 19% in retrospective studies.^17^ In these cases, there was no difference demonstrated according to public versus private hospital, or regional verses city location of residence. We can only speculate as to the reason for these differences in our cohorts; however, influencing factors may be surgical waiting times, resource allocation, differences in imaging capabilities, and surgeon practice.

**FIGURE 1:**
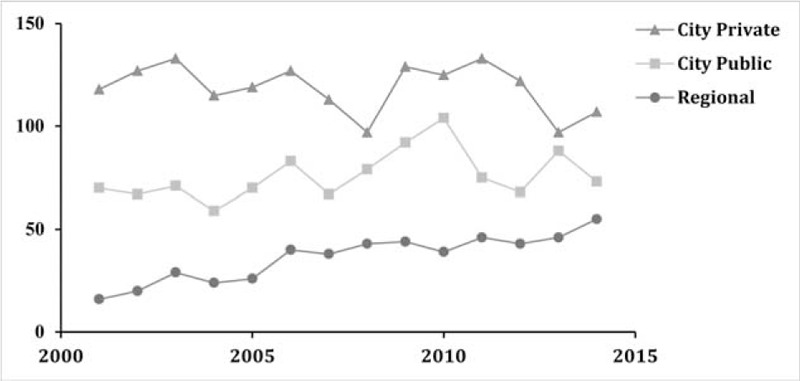
Total intracranial neoplasms per year in 3 NSW hospitals between 2001 and 2015. NSW = New South Wales.

Over the study period, although the caseload remained constant in city-based centers, there has been a steady growth of the neuro-oncology activity in the regional center (Fig. 1). Over the 14-year period of this study, new intracranial tumor diagnoses doubled at the regional center. This was likely a reflection in part of the addition of a second neurosurgeon to the department in 2004, and a third in 2005. Regional population growth and differences in referral patterns may be a further factor in the increased surgical activity of the regional center, as the number of cases continued to climb even at the study's conclusion. The recent rise in the activity of the regional center may be related in the increased public and general practice awareness of the department's knowledge, expertise, and skills through public education presentations, and news media releases. In addition, there is an increased trend for patients to prefer receiving treatment in centers close to their residence.

## CONCLUSIONS

We report the characteristics of a database comprised of over three and a half thousand histologically defined brain tumors gathered from public and private, city, and regional settings, over an 11-year period in Australia. This regional center had a higher proportion of intracranial metastatic disease, and less meningeal disease compared with the city center. There was also a lower rate of reoperation for metastatic disease in the regional center. In addition, in the regional center, there were fewer pathological diagnoses of tumors with oligodendroglial differentiation. Whereas the neuro-oncology case load is smaller for this regional center, the number of cases have steadily increased throughout the study period, and it is likely that more neuro-oncology surgery will be undertaken in regional centers in the future.
